# High-Fidelity Simulation for Assessing Catheterization-Based Skills Among Trainees

**DOI:** 10.1016/j.jscai.2026.105499

**Published:** 2026-06-30

**Authors:** Nicholas B. Mitchell, Luai Madanat, Shazil Mahmood, Justin Armstrong, Michael Gallagher, Robert D. Safian, Ryan D. Madder

**Affiliations:** Center for Innovation & Research in Cardiovascular Diseases, Department of Cardiovascular Medicine, William Beaumont University Hospital, Corewell Health East, Royal Oak, Michigan

**Keywords:** coronary angiography, medical education, simulation

## Abstract

**Background:**

Simulation-based training for coronary angiography has not been widely adopted, and an operator’s skillset should be accurately reflected in a simulation-based task. The purpose of this study is to determine whether increasing levels of clinical experience were associated with improved performance and whether trainees’ confidence improved with use of a high-fidelity simulator.

**Methods:**

Physicians with different catheterization experience (internal medicine residents, first- through third-year cardiology fellows, interventional fellows, and attending interventional cardiologists) performed multiple coronary artery catheter engagements on a high-fidelity endovascular simulator. The primary outcome was coronary engagement time (from the ascending aorta to coronary). Trainees completed presimulation and postsimulation surveys assessing confidence in catheter engagement (10-point Likert scale: 1, no confidence; 10, supreme confidence).

**Results:**

Across 240 catheter engagements by 24 physicians, median coronary engagement time was 37 seconds (IQR, 22-64). Engagement times were longer on the right coronary artery (42 seconds [22-70]) than on the left (34 seconds [21-56]; *P* = .04). Engagement time decreased with increasing operator experience (*P*_trend_ < .001). Baseline confidence increased with training stage (*P*_trend_ < .001), and among all trainees combined, confidence in catheter engagement improved after simulation (6 [2-7] vs 7 [5-8]; *P* = .01).

**Conclusions:**

Using a high-fidelity simulator, increasing operator experience was associated with less time to coronary catheter engagement. Simulation was associated with a significant increase in trainee confidence in performing coronary catheter engagement.

## Introduction

Simulation-based training has been integrated into several aspects of medical education, offering a safe and controlled environment for learners to develop technical skills, build confidence, and standardize educational experiences.^1^ In procedural skill acquisition, such as coronary angiography, simulation has the potential to accelerate skill acquisition and increase operator confidence, without introducing risk to patients and without radiation exposure. Despite these advantages, the integration of simulation into cardiology training programs has been limited.^2^^,^^3^ Current guidelines recommend its use,^4^^,^^5^ but widespread adoption has lagged partially due to a lack of supporting evidence.

This study was designed to evaluate high-fidelity simulation for coronary angiography. For simulation to be an effective training tool, it should reflect an operator’s skillset and experience in the simulation-based task, such that less experienced operators would complete the task less effectively than more experienced operators. The purpose of this study is to determine whether increasing levels of coronary angiography experience were associated with improved performance during simulated coronary artery catheter engagement and whether trainees’ confidence improved after performing catheterization on a high-fidelity simulator.

## Materials and methods

A total of 24 physicians volunteered to participate in the study. Participants included physicians with different levels of coronary angiography experience, including internal medicine residents; first-, second-, and third-year clinical cardiology fellows; interventional cardiology fellows; and attending interventional cardiologists. Each participant completed a presimulation survey to assess their confidence in coronary artery catheter engagement. This survey was followed by a brief tutorial on the functionality of a high-fidelity endovascular simulator. Participants with <1 month of coronary angiography experience were provided with a short tutorial on cardiac landmarks and the technique of coronary artery catheter manipulation. This study did not enroll patients, so approval by the institutional review board was not required.

### Endovascular simulator and coronary artery engagement

**Figure 1:**
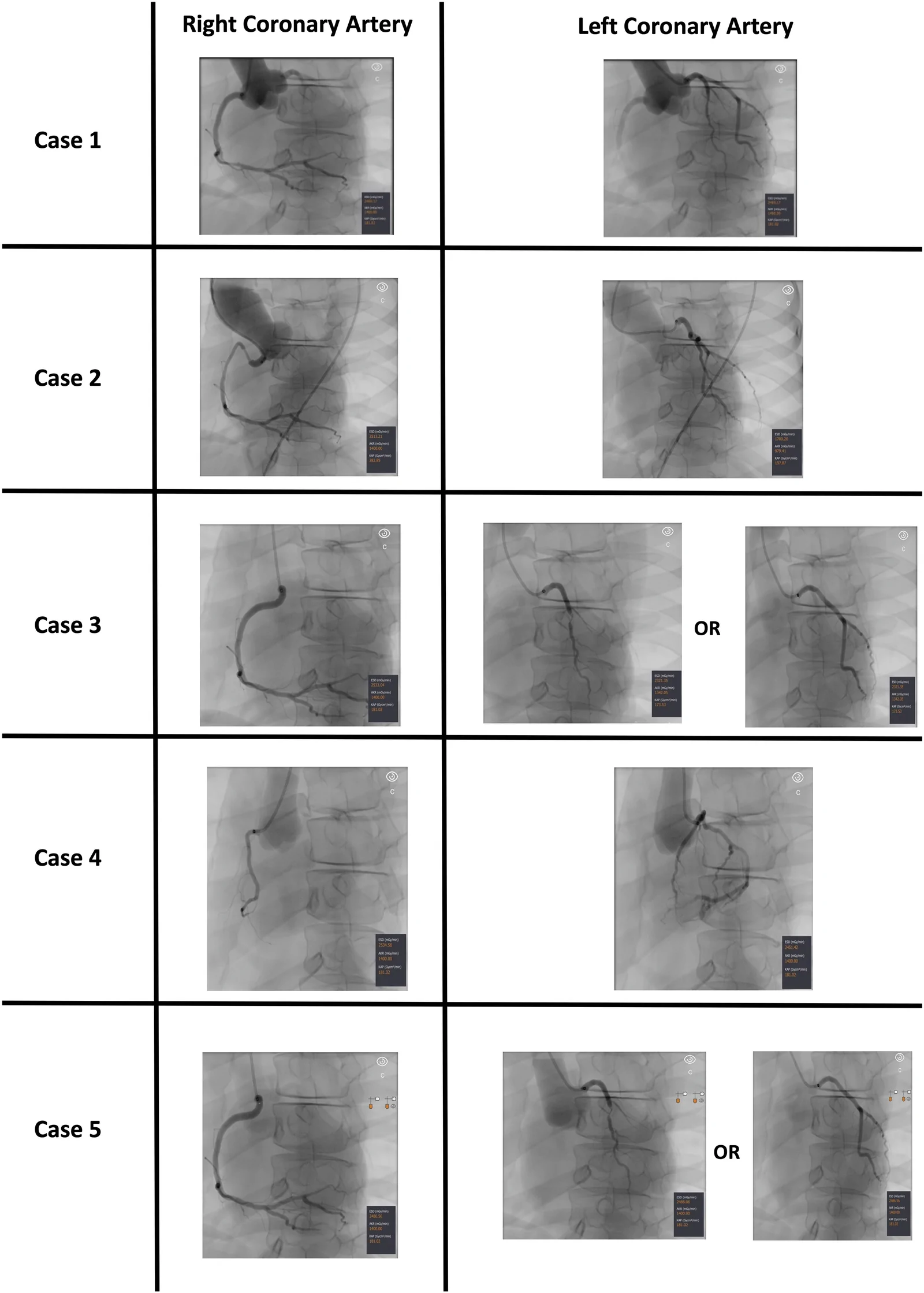
**Simulated cases of coronary angiography.** All cases were completed in the left anterior oblique projection at 30°.

All procedures were performed on a high-fidelity endovascular simulation system (VIST G7; Mentice), which simulates real-time endovascular procedures. The simulator has an access site into which actual interventional devices, such as guide wires and catheters, are inserted. After insertion, catheter advancement, retraction, and rotation are detected by sensors in the simulator and displayed on a virtual fluoroscopic monitor of a simulated patient. The system software allows simulated coronary artery engagement in patients with different coronary artery anatomy, coronary artery takeoffs, and ascending aortic configurations (Figure 1). Each simulated case requires the operator to advance, retract, and rotate real interventional devices in a manner akin to in vivo coronary artery engagement.

### Coronary artery engagement

Each study participant completed 5 consecutive simulation cases in which they attempted to engage diagnostic catheters into the ostia of the right and left coronary arteries. Simulated cases differed by coronary artery anatomy, location of the coronary takeoff, and aortic configuration. Attempts were performed from the right radial approach in 4 of the simulations and the right femoral approach in 1 of the simulations. All attempts started with a 0.035-inch J-tipped guide wire in the coronary cusp and a diagnostic catheter in the ascending aorta at the level of the second rib. After advancing the catheter into the aortic root, the operator retracted the guide wire and attempted to engage the tip of the catheter into the coronary ostium. Diagnostic catheters were selected by study personnel to maintain consistency across all participants. All coronary engagements were attempted with simulated real-time fluoroscopy with the gantry in the left anterior oblique 30° projection. The coronary catheter configuration to be used in each case was preselected by study personnel. Cases 1, 3, 4, and 5, which required right radial access, were performed using a Judkins Right 4 and Judkins Left 3.5 for right and left coronary engagement, respectively. Case 2 was performed from a right femoral approach with a Judkins Right 4 and Judkins Left 4 for right and left coronary engagement, respectively. Study personnel provided simulated contrast injections upon request from the study participant during attempts to engage the target artery.

### Study endpoints

The primary end point was catheter engagement time, defined as the time required to advance the catheter from its starting point to engagement of the catheter tip in the target coronary artery. The secondary end point was physician confidence with coronary engagement. Physician confidence was assessed before and after simulation, using a 10-point Likert Scale ranging from 1 (no confidence) to 10 (supremely confident).

### Statistical analysis

Continuous variables are presented as mean ± SD or as median with IQR, based on the presence or absence of a normal distribution, respectively. Normality was assessed using the Shapiro–Wilk test. Nonparametric comparisons were performed using the Mann–Whitney *U* test. Linear trends across ordered groups were evaluated using 1-way analysis of variance with linear polynomial contrasts, and corresponding *P* values were reported for the trend. All tests were 2-sided, and a *P* value of <.05 was considered statistically significant. Statistical analyses were performed using R software, version 4.0.4 (R Foundation for Statistical Computing).

## Results

A total of 24 physicians with catheterization experience levels ranging from novice to expert participated in the study. Trainee procedural experience, including months of training in the catheterization laboratory and years since medical school graduation, are depicted in the Central Illustration.

**Central Illustration:**
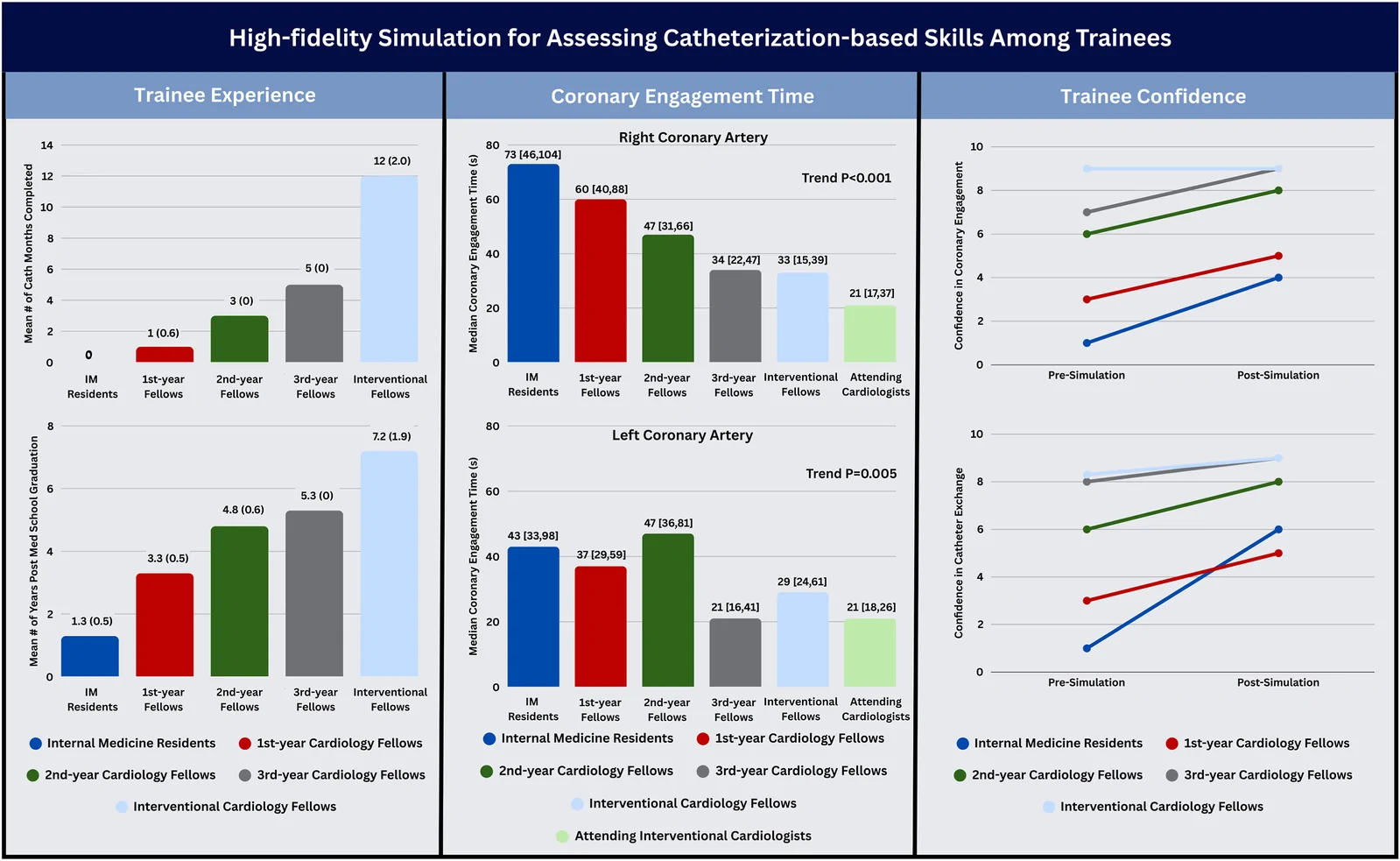
**High-fidelity simulation for assessing catheterization-based skills among trainees.** The left panel displays median number of years post medical school graduation for the 6 cohorts of the study. The middle panel displays the median coronary engagement times stratified by level of experience. The right panel shows the median change in operator confidence with coronary engagement and catheter exchange.

### Simulated coronary engagement

A total of 240 attempts to engage a catheter into a target coronary artery were collectively made by the 24 physicians. Median coronary engagement time was 37 seconds (IQR, 22-64); time to engage the right coronary artery (42 seconds [22-70]) was significantly longer than the time to engage the left coronary artery (34 seconds [21-56]; *P* = .04).

**Figure 2:**
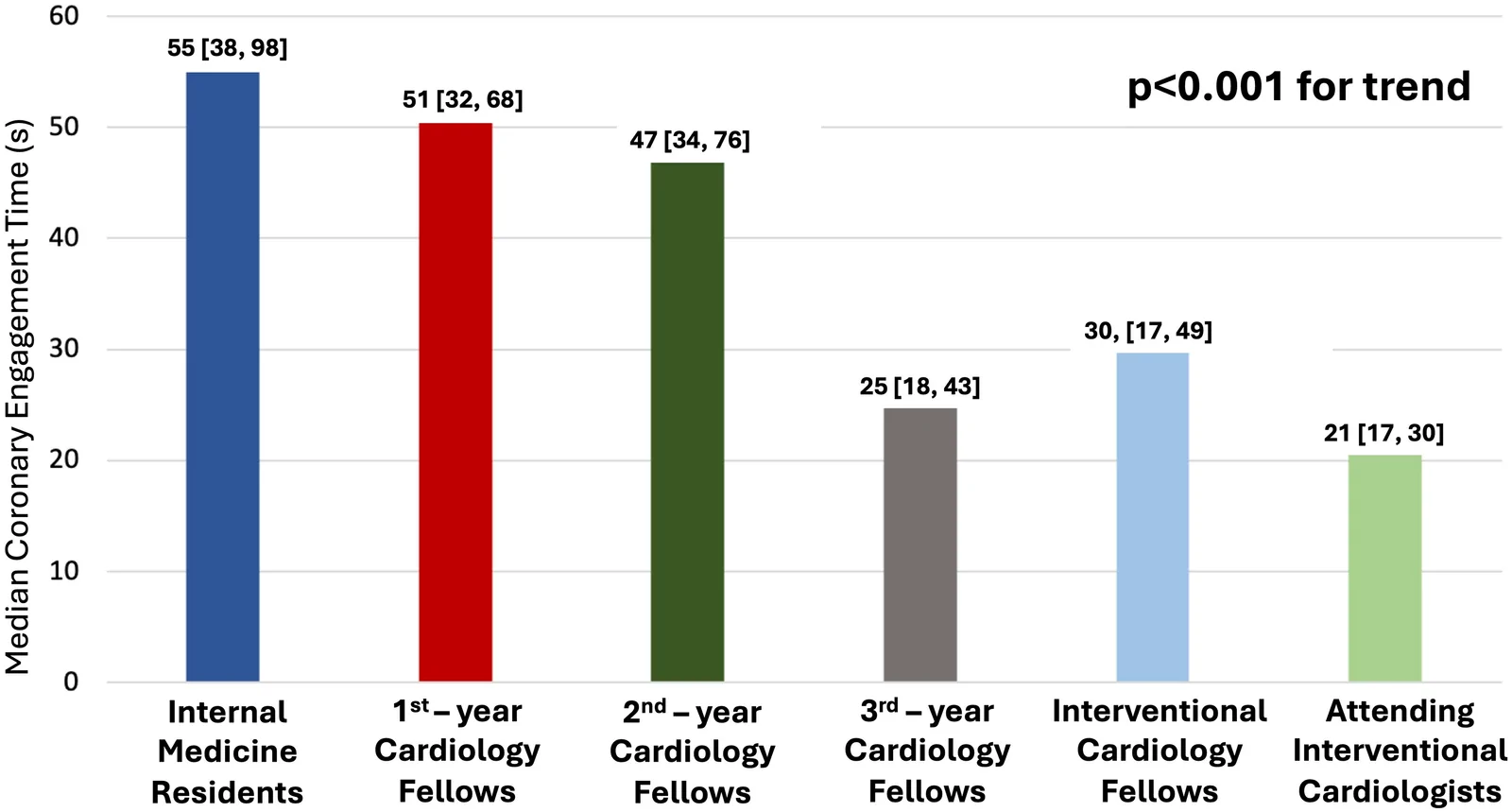
**Median coronary engagement time according to operator experience.** Shown are median times to engage a catheter into a target coronary artery on a high-fidelity simulator among physicians with various levels of catheterization experience. Interquartile ranges are shown in brackets.

Median coronary engagement times according to physician operator experience are shown in Figure 2 and Table 1. A significant trend was observed wherein coronary engagement time decreased with increasing level of physician experience (*P*_trend_ < .001). Increasing physician experience was associated with a significant trend toward faster coronary engagement times for both the right (*P*_trend_ < .001) and left (*P*_trend_ < .005) coronary arteries (Central Illustration). Internal medicine residents, first-year fellows, and second-year fellows had significantly longer coronary engagement times than attending interventional cardiologists (Table 1). Engagement times for third-year and interventional fellows were not significantly different than those of attending interventional cardiologists.

**Table 1:** Coronary engagement times according to physician experience level

Experience level	Coronary engagement time, s	*P* value
Interventional cardiologist	21 (17-30)	Reference
Interventional fellow	30 (17-49)	.13
Third-year cardiology fellow	25 (18-43)	.22
Second-year cardiology fellow	47 (34-76)	<.001
First-year cardiology fellow	51 (32-68)	<.001
Internal medicine resident	55 (38-98)	<.001

Coronary engagement times are shown as median (IQR). *P* values are for comparisons of engagement times to the engagement times of interventional cardiologists as the reference standard.

### Operator confidence

Self-reported trainee confidence levels before and after simulation are shown in the Central Illustration. Prior to simulation, confidence was lowest among internal medicine residents and increased significantly across trainee experience levels (*P*_trend_ < .001). Among all trainees combined, overall confidence levels significantly increased presimulation vs postsimulation for catheter engagement (6 [2-7] vs 7 [5-8]; *P* = .01).

## Discussion

In this study, we report several notable findings regarding basic cardiac catheterization procedural skills assessed using a high-fidelity endovascular simulator. First, we observed a significant trend in coronary catheter engagement time with physician experience level, such that increasing levels of procedural experience were associated with more efficient task completion. The trends toward faster catheter engagement with increasing operator experience were significant with attempted engagement of both the right and left coronaries. These observations are consistent with those of prior studies showing a graded increase in performance with increasing physician experience.^6^^,^^7^ Although the present observations suggest contemporary high-fidelity simulators may be capable of determining the catheterization skill level of a trainee, future studies will be required to determine whether catheterization skill level can be advanced through repetitive simulation. It should be noted that repetitive simulation has been shown to improve technical skill development among surgical trainees.^8^ Second, we observed the simulation experience to be associated with significant increases in trainee confidence levels in performing catheter engagement. Additional studies are needed to determine whether this improvement in confidence through simulation will translate into reduced procedural anxiety and improved performance among trainees when performing catheterization in patients.

### Simulation of catheter engagement

One of the most technically challenging procedural competencies for cardiology fellows to master when learning coronary angiography is catheter engagement into the right coronary artery. Unlike engagement of a catheter into the left coronary artery, which often requires minimal catheter manipulation, engagement of the right coronary artery requires considerably greater catheter torquing and maneuvering to transmit the applied torque. Consistent with this premise, we observed a longer median catheter engagement time for the right coronary artery than for the left when using the simulator. It is also notable that the median catheter engagement time of the right coronary artery was 73 seconds among novices and only 21 seconds among interventional cardiologists. In contrast, engagement of the left coronary artery among novices was 43 seconds and among interventional cardiologists was 21 seconds, a much small difference than observed with right coronary artery engagement. These observations suggest the high-fidelity simulator appropriately captured the greater difficulty trainees experience when learning to engage the right coronary artery compared with the left.

Another interesting observation regarding catheter engagement times was that internal medicine residents, first-year cardiology fellows, and second-year fellows each had significantly longer engagement times than interventional cardiologists, whereas engagement times for third-year and interventional cardiology fellows were not significantly different than those of interventional cardiologists. This suggests simulation-based training of coronary catheter engagement may have diminishing returns as trainees transition through fellowship. Hence, the best period of training in which simulation of coronary artery engagement should be applied to accelerate mastery of this competency is early in training. Considering contemporary endovascular simulators offer simulations of procedures considerably more complex than coronary angiography, additional study will be required to determine how accurately simulations of percutaneous coronary interventions, structural interventions, and peripheral interventions reflect trainee skillsets.

### Current role of simulation in cardiology fellowships

Simulation-based training has been endorsed by major societies such as the Society for Cardiovascular Angiography and Interventions, the American College of Cardiology, and the American Board of Internal Medicine.^4^^,^^9^^,^^10^ Simulation has been studied in the context of basic clinical competencies such as transvenous pacemaker placement, pericardiocentesis, and intra-aortic balloon pump placement and demonstrated to result in improvement in both written and technical competencies.^11^ Simulation has also been studied to determine effectiveness in teaching transesophageal echocardiography, with shown improvement in knowledge, skills, and proficiency.^12^ In terms of coronary angiography, a small prior endovascular-based research study demonstrated gains in the confidence and technical skills of 7 first-year cardiology fellows when using endovascular simulation.^13^ Our study is consistent with these prior results by affirming the association of simulator use with increased trainee confidence and extends the prior findings by demonstrating the association of clinical experience with performance outcomes on a high-fidelity simulators.

Despite the demonstrated advantages of simulation in cardiology training and the benefits shown across multiple studies, there continues to be a lack of widespread simulation adoption within cardiology training programs. We postulate this may be attributable to insufficient financial resources among many fellowship programs to purchase a high-fidelity simulator. Acquisition of simulation equipment and software takes both financial commitments from training institutions and the breaking of the status quo in which direct experience on patients is the mainstay of clinical training.

### Limitations

This study is limited by its observation design, relatively small sample size, and conduct at a single center. Since all trainees in this study were from the same institution, additional studies are needed to determine whether similar findings are observed at other centers. This study was designed to evaluate whether existing skill-level prior to the use of simulation would be captured accurately in a single session using a high-fidelity simulator. A limitation of the study is that trainees did not participate longitudinally in multiple sessions in order to measure changes in skill level over time. Furthermore, whether trainee competence on the simulator translated into skills performing catheterization on actual patients was not assessed, and this represents another limitation of the study design. Considering the majority of cases were performed from the radial access approach, the lack of a larger number of femoral access cases represents another limitation. The study is further limited in that catheters for each case were preselected by study personnel. Additional studies are needed to determine whether simulation can teach trainees to select an appropriate catheter for a given anatomy. Another limitation of this study is the limited number of trainees in each group. Larger studies are needed to assess the statistical significance of increasing operator confidence within each trainee subset. Finally, the study is limited since other catheterization-based skills, such as table panning and selection of gantry angle, were not studied.

## Conclusion

Performance of various catheter-based skills on a high-fidelity simulator were observed to trend significantly with physician operator procedural experience. Use of a simulator was also associated with a significant increase in trainee confidence level in performing catheter engagement and catheter exchanges.

## CRediT authorship contribution statement

**Nicholas B. Mitchell:** Writing – review & editing, Writing – original draft, Validation, Supervision, Investigation, Formal analysis, Data curation, Conceptualization. **Luai Madanat:** Formal analysis, Data curation, Conceptualization. **Shazil Mahmood:** Methodology, Data curation, Conceptualization. **Justin Armstrong:** Visualization, Software, Data curation. **Michael Gallagher:** Writing – review & editing. **Robert D. Safian:** Writing – review & editing, Writing – original draft. **Ryan D. Madder:** Writing – review & editing, Writing – original draft, Supervision, Software, Methodology, Funding acquisition, Formal analysis, Conceptualization.

## Declaration of competing interest

Ryan D. Madder has received speaker honoraria from Abbott Vascular, ACIST, Boston Scientific, and Corindus; has served as a consultant to Abbott Vascular, ACIST, AngioWave Imaging, Boston Scientific, MiRus, Nanoflex Robotics, Orchestra Biomed, RapidAI, and SpectraWAVE; has received research support from AngioWave Imaging, Corindus, Microbot Medical, and Nanoflex Robotics; and serves on the advisory boards of Boston Scientific, Gentuity, Medtronic, and SpectraWAVE. Robert D. Safian is a consultant for HeartFlow and is on the advisory board for HeartFlow and Medtronic. Michael Gallagher is a consultant for HeartFlow. Nicholas B. Mitchell, Luai Madanat, Shazil Mahmood, and Justin Armstrong reported no financial interests.

## References

[1] Elendu C., Amaechi D.C., Okatta A.U. (2024). The impact of simulation-based training in medical education: a review. Medicine (Baltimore).

[2] Lee K.S., Klein A.J., Bricker R.S. (2025). Current state of simulation in interventional cardiology training: results of a SCAI survey. J Soc Cardiovasc Angiogr Interv.

[3] Bagai A., O’Brien S., Al Lawati H. (2012). Mentored simulation training improves procedural skills in cardiac catheterization: a randomized, controlled pilot study. Circ Cardiovasc Interv.

[4] Green S.M., Klein A.J., Pancholy S. (2013). The current state of medical simulation in interventional cardiology: a clinical document from the Society for Cardiovascular Angiography and Intervention’s (SCAI) Simulation Committee. Catheter Cardiovasc Interv.

[5] Westerdahl D.E. (2016). The necessity of high-fidelity simulation in cardiology training programs. J Am Coll Cardiol.

[6] Lee K.S., Natarajan B., Wong W.X. (2022). A randomized controlled trial of simulation training in teaching coronary angiographic views. BMC Med Educ.

[7] Lipner R.S., Messenger J.C., Kangilaski R. (2010). A technical and cognitive skills evaluation of performance in interventional cardiology procedures using medical simulation. Simul Healthc.

[8] Woodall W.J., Chang E.H., Toy S. (2024). Does extended reality simulation improve surgical/procedural learning and patient outcomes when compared with standard training methods? A systematic review. Simul Healthc.

[9] Baldassarre L.A., Mendes L.A., Blankstein R. (2026). 2025 ACC/AHA/ASE/ASNC/SCCT/SCMR advanced training statement on advanced cardiovascular imaging: a report of the ACC Competency Management Committee. J Am Coll Cardiol.

[10] American Board of Internal Medicine (2018). Policies and Procedures for Certification. American Board of Internal Medicine.

[11] Young M.N., Markley R., Leo T. (2018). Effects of advanced cardiac procedure simulator training on learning and performance in cardiovascular medicine fellows. J Med Educ Curric Dev.

[12] Pezel T., Dreyfus J., Mouhat B. (2023). Effectiveness of simulation-based training on transesophageal echocardiography learning: the SIMULATOR randomized clinical trial. JAMA Cardiol.

[13] Casey D.B., Stewart D., Vidovich M.I. (2016). Diagnostic coronary angiography: initial results of a simulation program. Cardiovasc Revasc Med.

